# Two-Stage Categorization in Brand Extension Evaluation: Electrophysiological Time Course Evidence

**DOI:** 10.1371/journal.pone.0114150

**Published:** 2014-12-01

**Authors:** Qingguo Ma, Cuicui Wang, Xiaoyi Wang

**Affiliations:** 1 School of Management, Zhejiang University, Hangzhou, The People's Republic of China; 2 Neuromanagement Lab, Zhejiang University, Hangzhou, The People's Republic of China; 3 School of Management, Hefei University of Technology, Hefei, The People's Republic of China; Southwest University, China

## Abstract

A brand name can be considered a mental category. Similarity-based categorization theory has been used to explain how consumers judge a new product as a member of a known brand, a process called brand extension evaluation. This study was an event-related potential study conducted in two experiments. The study found a two-stage categorization process reflected by the P2 and N400 components in brand extension evaluation. In experiment 1, a prime–probe paradigm was presented in a pair consisting of a brand name and a product name in three conditions, i.e., in-category extension, similar-category extension, and out-of-category extension. Although the task was unrelated to brand extension evaluation, P2 distinguished out-of-category extensions from similar-category and in-category ones, and N400 distinguished similar-category extensions from in-category ones. In experiment 2, a prime–probe paradigm with a related task was used, in which product names included subcategory and major-category product names. The N400 elicited by subcategory products was more significantly negative than that elicited by major-category products, with no salient difference in P2. We speculated that P2 could reflect the early low-level and similarity-based processing in the first stage, whereas N400 could reflect the late analytic and category-based processing in the second stage.

## Introduction

Brand recognition is a special social cognition phenomenon in modern commercialized society. A brand name can be considered an artificial mental category, which is used to distinguish the products of one company from those of another by showing their special qualities [1]–[3]. Brand extension uses an existing brand name to sell a new product [1]. When consumers encounter the branded new product, they evaluate the relationship between the brand and the product in terms of attribute similarity [3]. For example, Pepsi is a famous brand with many soft drink and food products, but it cannot extend to household appliances. Brand extension evaluation can be considered a classification and categorization process [4]. Marketing practitioners often manage the products of their companies by brand extension for economic and strategic reasons. Brand extension provides an opportunity to study the cognitive process of categorization.

Categorization theory suggests that individuals place objects in different categories to understand and process them well [5]. Similarity-based categorization is a representative categorization process that involves the comparison of a test object with recalled examples of the category or with a mental prototype that represents category members [6]–[8]. That is, similarity to a prototype or all previous examples determines whether the object belongs to the category. In marketing, consumers consider the brand as a salient cue to classify existing products [9]. If a new product is included in a known brand, consumers judge the product by a categorization process in which extension evaluation is determined by the perceived category overlap between the new member and the brand attributes [10]–[12].

Smith et al. explained categorization as a two-stage process in which decisions about logical matters in the first part are quickly made on the basis of overall similarity and those in the second part involve a deliberative process [13]. Literature on brand extension suggests that consumers consider not only information on product-level feature similarity between the new product and the products already associated with the brand but also the consistency between the brand concept and the extension [3]. We speculate that brand extension includes at least two stages related to similarity-based categorization by time course. Evidence from hemodynamic imaging indicated that the anterior prefrontal and posterior cingulate regions significantly affect similarity-based categorization [14]. However, no direct neurological evidence has been found from the perspective of the electrophysiological time course to study the process of categorization. We used event-related potential (ERP), a non-invasive brain-scan technique, to study the specific process of similarity-based categorization in brand extension. Moreover, the research can facilitate the study of the cognitive and affective responses of consumers to marketing stimuli and help marketing researchers to explore the mechanism of why consumers make the decisions they do, and to develop new products and services effectively.

Two ERP components have been associated with the processing of categorization, namely, P2 and N400. P2 is a positive potential over frontal regions that presumably reflects the early assessment of stimuli and is generated by the orbitofrontal cortex [15]. P2 as an independent ERP component indicates more automatic perceptive activity [16],[17].

Many studies have associated P2 with low-level word identification processes [18]. Thomas et al. (2007) found that threat words evoke larger P2 amplitudes than neutral words, suggesting that an emotional word can be distinguished at early stages of attention during the low-level processing of stimuli [19]. In the process of perception and evaluation for warning signal word, P2 reflected the engagement of attention resources and associated with the detection of hazard for them [20]. In the study of visual selective attention, the P2 findings may further suggest that this component reflected perceptual rather than post-perceptual processing [21]. More and more ERP studies had also indicated that P2 was involved in semantic processing. Blanchet et al. (2007) found that the P2 reflected the attention processes that increase with the organizational semantic demand [22]. Freunberger et al. (2007), using a paradigm ensured a top-down activation of semantic categories, indicated that the P2 reflected the top-down regulation processes with smaller amplitudes for congruent compared to incongruent targets [23]. Lei et al. (2010) also found that the P2 amplitude of atypical members was larger than that of typical members, which was related to the process of the participants' early detection of an item's category membership [24]. In a study of the evaluation of the task relevance of visual stimuli, Potts found that P2 is present with task-relevant stimuli but has the same scalp topography and estimated source-dipole locations in overt and covert responses, indicating stimulus evaluation rather than response production [25]. These studies have shown that P2 can reflect the rapid and automatic assessment of the early stage of stimulus similarity between different categories of low cognitive levels, followed by the progressive recruitment of slow, elaborative, and semantic processing under voluntary control. We inferred that distant branded extensions as new and unpredictable stimuli elicit larger P2 (positive polarity) than others.

Kutas and Hillyard (1980) found that N400 is a negative ERP component that peaks at approximately 400 ms after the presentation of a word or picture. The amplitude of N400 is affected by many factors, such as relatedness to a preceding item, congruence (context-appropriateness) of sentence endings, and strength of word association [26]–[28]. The amplitude is large when the processes are difficult [29]. Kutas and Federmeier (2000) used a sentence verification paradigm and found that N400 amplitudes decrease when the exemplar is a member of the category (e.g., a carrot is a vegetable) [30]. Polich (1985) demonstrated that N400 is elicited when the presented word conflicts with a semantic category not only in sentences but also in pairs of words [31]. In category priming paradigms, such as “pear/pear” (exact match), “pear/apple” (in-category), and “pear/curtain” (out-of-category), out-of-category words yield a more significantly negative N400 than others. Similarly, in indirect priming paradigms, such as “birthday/cake” (directly primed), “birthday/pie” (indirectly primed), and “birthday/soap” (unprimed), the N400 elicited by indirectly primed and unprimed pairs is more significantly negative than that elicited by directly primed pairs [32]. The semantic typicality of an exemplar modulates the amplitude of the N400 component, and both atypical members (in-category) and nonmembers (out-of-category) elicit a large (significantly negative) N400 [33],[34]. In many studies, participants focused their attention on the stimuli, behavior that is vital to N400 elicitation. However, a similar effect on N400 was observed with no required attention to the semantic relationship between words [35]. N400 following P2 reflected more classification attempts among different categories than early components, possibly representing different stages in the categorization process.

Most studies in marketing have argued that the evaluation of brand extension is a conscious process [1],[3],[4]. Ma et al. (2008) found that conscious categorization processing in brand extension evaluation elicits P300 in a decision task of whether to accept or reject the brand extension [36]. However, P300 is widely responsive to decision making [37], and Ma et al. did not distinguish the categorization process from category-related decision making. Wang et al. (2012) used a non-related task to study the beverage brand extension into clothing products and demonstrated that N400, not P300, reflects uncontrolled categorization in brand extension [38].

In our study, we also used beverage brands as the prime stimuli but manipulated more conditions, namely, out-of-category extension (distantly extends to the household appliance category), similar-category extension (closely extends to the snack category), and in-category products (no extension into the beverage category). With non-related tasks in brand extension evaluation, out-of-category extensions are distinguished at the early stage. In the late stage, close extensions with many similar attributes to those of the parent brand category are separated from the no-extension condition. Early processing is related to the similarity (low level) between new products and the products in the brand category; this similarity may be reflected by the early components of ERP. Later analytic processing may be concerned with the effects of conflicting information when different products are compared with prototype products in the brand category. This stage may be reflected by the later components of ERP. We speculated that P2 and N400 would be observed and would reflect different stages in the categorization process in the present study. To further support the P2 and N400 effects in the categorization theory of brand extension, we designed experiment 2 with the same brand names extended to subcategory product names (e.g., cookie and bread) and major-category product names (e.g., dessert and snack). Experiment 2 aimed to provide evidence that N400, not P2, reflects the high-level, analytic, and elaborative categorization process from another perspective of categorization theory.

## Materials and Methods

This study was approved by the Internal Review Board of Zhejiang University Neuromanagement Lab. Before both experiments were formally started, written informed consents were obtained from participants.

### 1 Participants

Eighteen (eight females, all right-handed according to their accounts) and 14 (six females, all right-handed) non-business-major undergraduates from Zhejiang University participated in experiments 1 and 2, respectively, as paid volunteers. In experiment 1, the mean age of the participants was 22.5 years (range: 19–26 years). The data on one participant (male) were excluded because the number of valid trials (below 30) was insufficient. In experiment 2, the mean age of the participants was 24.5 years (range: 23–28 years). The participants were all native Chinese speakers and had normal or corrected-to-normal vision according to their self-reports. No participant had a history of neurological or psychiatric abnormalities.

### 2 Experiment 1

#### 2.1 Experimental stimuli in experiment 1

We used a prime–probe paradigm in experiment 1. The prime stimuli (S1) consisted of 20 soft drink brands. We used the soft drink brands and categories as experiment materials because they were familiar to most students. In the pretest, the top 50 soft drink brands were chosen from “Famous Brands in China”, published by the State Trademark Administration of China. A group of 35 participants from Zhejiang University rated a list of familiar (measured via familiar vs. unfamiliar binary measure) of these brand names. Twenty brand names were chosen from the list for more than 85% participants being familiar with them. Before the experiment proper, participants were screened by a special brand familiarity test to ensure that all of them were familiar with the brands, including Nestlé, Coca Cola, and Evian. In China, most brand names have other meanings in the noncommercial context. For example, “coca” means “tasty” in Chinese; thus, we added the word “brand” to each prime stimulus after the brand name to emphasize that S1 was a brand. The probe stimuli (S2) comprised 12 product names chosen from three product categories (four product names per category), namely, in-category products (beverage, non-extension), similar-category products (snack, close extension), and out-of-category products (household appliance, distant extension). Each picture was digitized to 150×200 pixels. In addition, the mean luminance level of the pictures was 186.17 cd/m^2^ (candela/square meter), with standard deviation 25.82 cd/m2, which was matched and unified during experiment 1. The stimuli consisted of 240 pairs of brand names (S1) and product names (S2), i.e., 20 beverage brand names ×3 categories ×4 product names.

After the experiment, the participants completed a questionnaire with 24 words (12 product names and 12 brand names). Six of these words were selected from the prime stimuli, six from the probe stimuli (two product names per category from the probe stimuli), six from the new beverage brand names, and six from the new product names (two product names per category but different from the probe stimuli).

#### 2.2 Procedure

The experiment comprised three blocks, each of which included 80 pairs of brand names (S1) and product names (S2). Every pair sequence was randomized. The presentation of all stimuli was controlled by a stimulus system (Stim2, Neurosoft Labs, Inc., Sterling, VA, USA). Each picture of stimuli was presented in the center of a screen with black word on gray background. Viewing distance was 1 m resulting in a horizontal and vertical visual angle of 2.58° and 2.4°.

**Figure 1:**
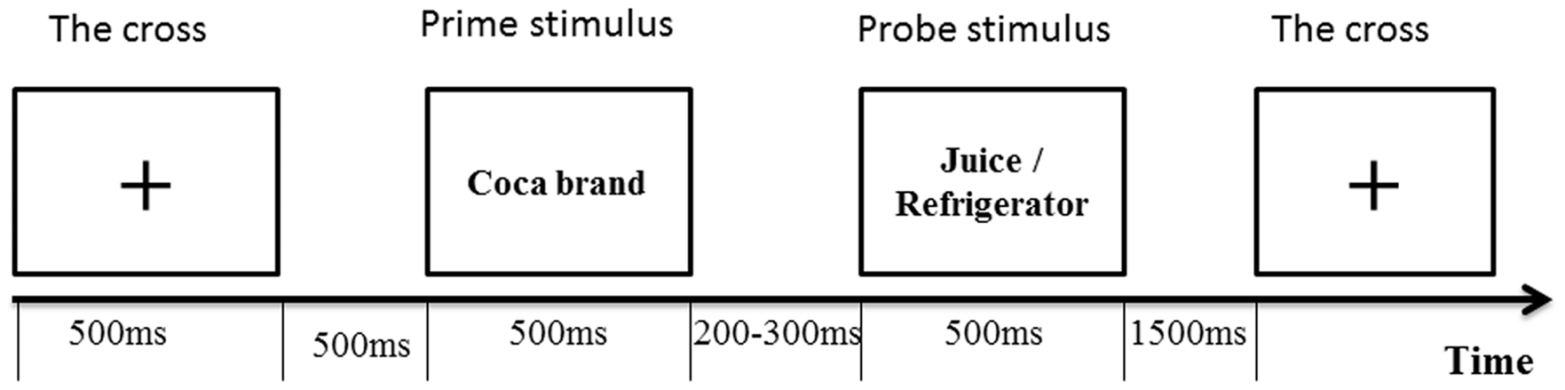
Experiment 1 procedure.

In each trial, every stimulus word was presented for 500 ms followed by a random inter-stimulus interval (ISI) between S1 and S2 that ranged from 200 ms to 300 ms (average  = 250 ms). The interval between the end of S2 and the onset of the following cross was 1500 ms (Fig. 1). The stimulus pictures were presented in the center of a computer screen, the presentation sequence of which was controlled by a stimulus system (Stim2, Neurosoft Labs, Inc., Sterling, Virginia, USA).

Participants were seated in front of a PC monitor and were required to fix their eyes on a cross at the center of the screen. The participants were instructed to remember S1 and S2, which would be tested after the experiment with a questionnaire. After the instructions, the participants completed 20 practice trials, which were excluded from the experiment materials. After the experiment, the participants were required to fill out a questionnaire to identify which name pairs were shown in the experiment.

The participants were paid 30 Chinese yuan (approximately US$ 4) as a basic payment. Additional monetary reward was given depending on their performance in completing the questionnaire. Such performance was one criterion used to examine whether the participants took the experiment seriously and to decide whether to exclude the participants from analysis. The prime–probe pairs (S1–S2) were randomly presented on the screen and had equal probability.

### 3 Experiment 2 procedure

In experiment 2, the prime stimuli (S1) were the same as those in the first experiment, consisting of 20 beverage brands. The probe stimuli (S2) comprised 12 product names chosen from snack and household appliance categories. In S2, six product names were subcategory product names, i.e., cookie, bread, chips, refrigerator, television, and air-conditioning, and the others were major-category product names, i.e., dessert, snack, dim sum, machine, household appliance, and electrical equipment. Each picture was digitized to 150×200 pixels. In addition, the luminance level of the pictures was unified and was the same with experiment 1. The stimuli consisted of 240 pairs of brand names (S1) and product names (S2), i.e., 20 beverage brand names ×12 product names (6 product names were subcategory product names, and the others were major-category product names).

The stimulus system employed to control the presentation of stimuli in experiment 2 was the same as that in the experiment 1. Each picture of stimuli was presented in the center of a screen with black word on gray background. In addition, the viewing distance, the horizontal and vertical visual angles were also the same as that in experiment 1. Experiment 2 adopted a similar manipulation to that in experiment 1, but participants were asked to judge whether a brand extension was suitable with the prime–probe paradigm (S1–S2 paradigm). The stimulus (S1 or S2) was always presented for fixation for 1000 ms each, and the ISI between S1 and S2 varied from 300 ms to 700 ms (average  = 500 ms). The interval between the end of the previous S2 and the onset of the following S1 was 2000 ms. The participants were paid 30 Chinese yuan (approximately US$ 4) as a payment.

### 4 Electroencephalogram recording

**Figure 2:**
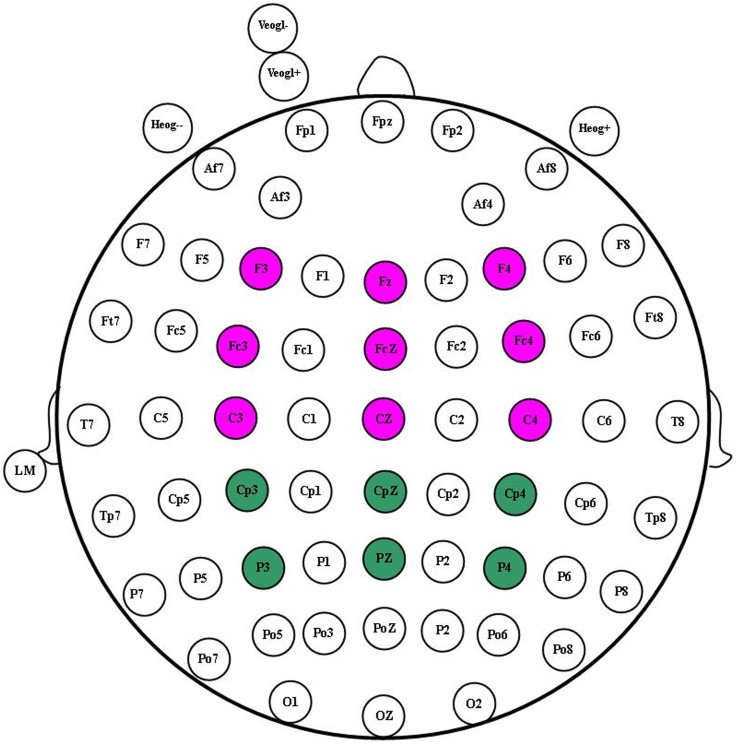
Electrode groups.

Both experiments were performed in an electrically shielded and soundproofed cabin. Participants sat in a comfortable chair. Electroencephalography (EEG) was recorded with a NeuroScan SynAmps2 amplifier (Scan 4.3.1, Neurosoft Labs, Inc.) with Ag/AgCl electrodes placed at 64 scalp sites according to the extended international 10–20 system and referenced to the left mastoid with a cephalic (forehead) location as the ground (see Fig. 2 for the recording sites). The band-pass was 0.05 Hz to 100 Hz at a sampling rate of 500 Hz. To detect blinks and vertical eye movements, vertical electro-oculograms (EOGs) were recorded with one pair of electrodes placed above and below the left eye, and horizontal EOGs were recorded with another pair 10 mm from the lateral canthi. Trials with ocular or movement artifacts were excluded from the average ERP waveforms. Electrode impedances were maintained below 5 kΩ throughout both experiments.

### 5 Electroencephalogram analysis

Offline EEG signal was processed by running a NeuroScan analysis software (Scan 4.5, Neurosoft Labs, Inc.). Electroencephalogram recordings were extracted from −200 ms to 800 ms time-locked to the onset of the probe stimulus (S2), with the pre-stimulus period as the baseline. EOG artifacts were corrected by the method proposed by Semlitsch et al. [39]. Through the subtraction of one half the activity recorded at the right mastoid from each sample of data recorded at each channel, the recordings were re-referenced offline to linked mastoid electrodes. Trials with electro-oculography activity or other artifacts (such as bursts of electromyographic activity and peak-to-peak deflection exceeding ±80 µV) were excluded from averaging. The remaining trials were digitally filtered with a low-pass filter at 30 Hz (24 dB/octave) and were corrected to the baseline (i.e., the pre-stimulus period). In experiment 1, the ERPs were averaged for every participant in each of the three conditions (beverage, snack, and household appliance categories). In experiment 2, the ERPs were averaged according to subcategory and major-category product names.

In both experiments, P2 was most prominent at the frontal and central sites. Thus, nine electrodes (F3, Fz, F4, FC3, FCz, FC4, C3, Cz, and C4 in the frontal, fronto-central, and central sites), which comprised the coronal and sagittal factors, were chosen for the P2 component (pink electrodes in Fig. 2). For the N400 component, 15 electrodes including the coronal and sagittal factors (F3, Fz, F4, FC3, FCz, FC4, C3, Cz, C4, CP3, CPz, CP4, P3, Pz, and P4) in the frontal, frontal–central, central, centro-parietal, and parietal areas were chosen (see Fig. 2 for the electrodes, including the pink and green ones). Subsequently, for the two experiments, a repeated-measures analysis of variance (ANOVA) was used to further analyze the effects of factors. To study the neurophysiologic features of the automatic evaluation of brand extension, a 3 (Product category) ×3 (Coronal) ×3 (Sagittal) within-subjects repeated-measures ANOVA for P2 and a 3 (Product category) ×5 (Coronal) ×3 (Sagittal) similar ANOVA for N400 were conducted in experiment 1. A 2 (Subcategory or Major-category product names) ×9 (Electrodes) ANOVA for P2 and a 2 (Subcategory or Major-category product names) ×15 (Electrodes) ANOVA for N400 were conducted in experiment 2. Greenhouse–Geisser [40] correction was used when necessary (uncorrected *df* was reported with the ε and corrected *p*-values), whereas Bonferroni correction was used for multiple paired comparisons. Only significant effects (*p*<0.05) are reported in detail.

## Results

### 1 Behavioral data

No behavioral data were recorded in experiment 1 because of the no-response task. The average accuracy rate of questionnaire recognition for the participants was 90.24%, with a standard deviation of 0.13. Only four participants identified one pair that never appeared in the experiment. If the accuracy rate of questionnaire recognition was greater than 80%, we considered the participant was serious and responsible during the experiment. We did not exclude any participant according to this criterion.

In experiment 2, the acceptance rate (AR) and reaction time (RT) were analyzed separately by a paired-sample test in subcategory and major-category products. AR is the rate of judging a brand extension as suitable. A significant difference in AR [*t* (13)  = 2.763, *p* = 0.016] was found, with no salient difference in RT [*t* (13)  = 0.558, *p* = 0.587]. The behavioral results are shown in Table 1.

**Table 1:** The Mean AR and RT (M±SD) in the experiment 2.

	AR	RT
Major-category product names	0.297±0.062	853.645±56.861
Sub-category product names	0.220±0.060	845.088±65.761

### 2 EEG data

#### 2.1 EEG data in experiment 1

**Figure 3:**
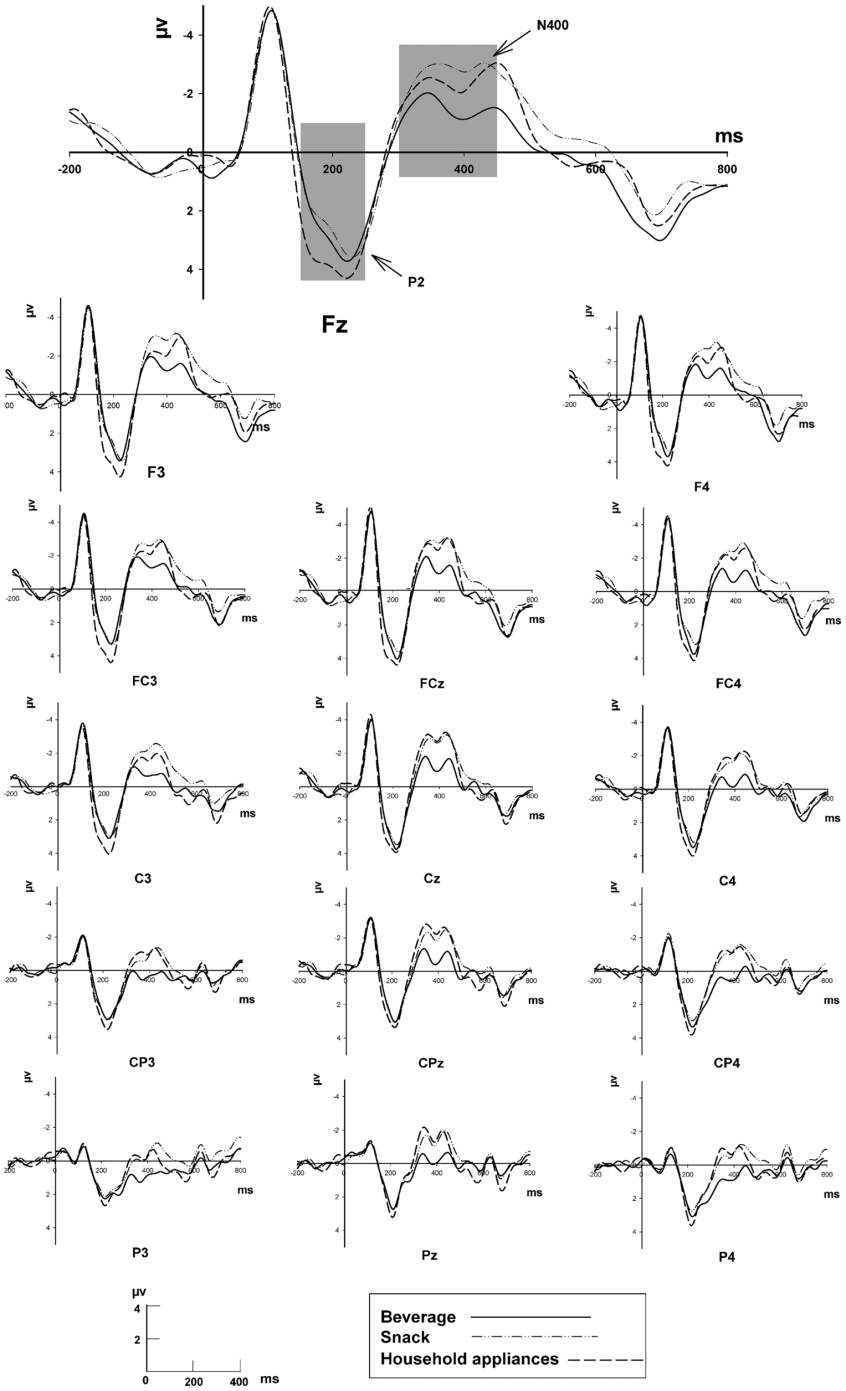
Grand-average ERPs elicited by three product categories with prime effect of beverage brand name at 15 electrodes in frontal, central, and parietal areas. Time window of 150 ms to250 ms for P2 quantification at F3, Fz, F4, FC3, FCz, FC4, C3, Cz, and C4, and time window of 300 ms to450 ms for N400 quantification at F3, Fz, F4, FC3, FCz, FC4, C3, Cz, C4, CP3, CPz, CP4, P3, Pz and P4 marked in light gray, respectively.

**Figure 4:**
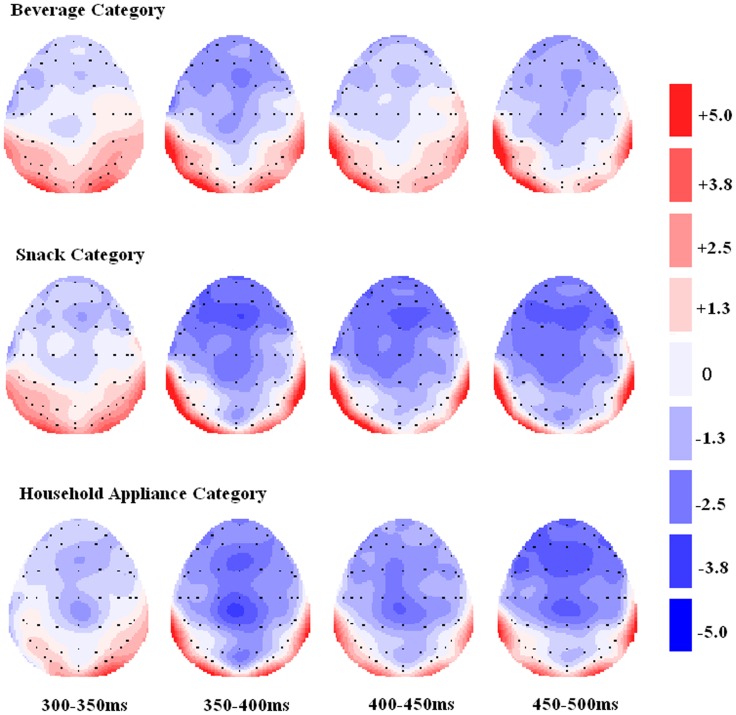
Topographic distribution of three product categories (beverage, snack, and household appliance).

The grand-average ERPs for the product categories of beverages, snacks, and household appliances are shown in Fig. 3. Fig. 4 shows the scalp distribution of the effects of the three categories (beverage, snack, and household appliance) at four time windows.

The ANOVA for the mean amplitude of P2 in the 150 ms to 250 ms time window (the raw data can be gotten from Table S1) revealed significant effects for Product category [*F* (2, 32)  = 7.71, *p* = 0.002] and Sagittal factors [*F* (2, 32)  = 3.62, *p* = 0.038]. However, no significant effects for Coronal factor were found [*F* (2, 32)  = 0.56, ε = 0.557, *p* = 0.481]. The Bonferroni-corrected pairwise comparison test showed that the mean amplitude of P2 elicited by the Product category of household appliances (3.6 µV) was significantly larger (positive polarity) than that by the categories of beverage (2.7 µV) (*p* = 0.009) and snack (2.4 µV) (*p* = 0.008). However, no significant difference was observed between the beverage and snack categories (*p* = 1.000). Although the pairwise comparison test for Sagittal factors showed that the mean amplitude of P2 across the electrodes at the midline (Fz, FCz, and Cz) was significantly different from the mean P2 amplitude across the left hemisphere (F3, FC3, and C3) (*p* = 0.035), no significant difference was found from that across the right hemisphere (*p* = 0.123). However, no significant hemisphere effect was observed, i.e., the mean amplitude of P2 (*p* = 1) in the left hemisphere was the same as that in the right. The ANOVA revealed that the extension Product category had no significant interaction with the Coronal [*F* (4, 64)  = 0.10, ε = 0.572, *p* = 0.388], Sagittal [*F* (4, 64)  = 1.60, *p* = 0.184], or Coronal × Sagittal [*F* (8, 64)  = 1.11, *p* = 0.361] factors.

The ANOVA for the mean amplitude of N400 in the 300 ms to 450 ms window (the raw data can be gotten from Table S2) found significant effects for the extension Product category [*F* (2, 32)  = 6.30, *p* = 0.005], Coronal factor [*F* (4, 64)  = 10.77, ε = 0.323, *p* = 0.002], and Sagittal factor [*F* (2, 32)  = 19.55, *p* = 0.000]. Bonferroni-corrected multiple paired comparisons revealed that the extension product in the beverage category yielded a significantly smaller (negative-polarity) amplitude of N400 (−0.6 µV) than the snack (−1.9 µV, *p* = 0.034) and household appliance categories (−1.8 µV, *p* = 0.019). No significant difference was observed between the extension product categories of snack and household appliances (*p* = 1). The ANOVA revealed that the extension Product category had no significant interaction with the Coronal [*F* (8, 128)  = 1.94, ε = 0.282, *p* = 0.153], Sagittal [*F* (4, 64)  = 2.13, *p* = 0.087], or Coronal × Sagittal [*F* (16, 256)  = 1.02, ε = 0.393, *p* = 0.416] factors.

Although the pairwise comparison test for the Sagittal factor showed that the mean amplitude of N400 across the electrodes at the midline (Fz, FCz, Cz, CPz, and Pz) was significantly different from those across the electrodes in the left hemisphere (F3, FC3, C3, CP3, and P3) (*p* = 0.000) and the right hemisphere (*p* = 0.000), no significant hemisphere effect was found, i.e., the left hemisphere did not differ from the right in terms of the mean amplitude of N400 (*p* = 1.000).

#### 2.2 EEG data in experiment 2

**Figure 5:**
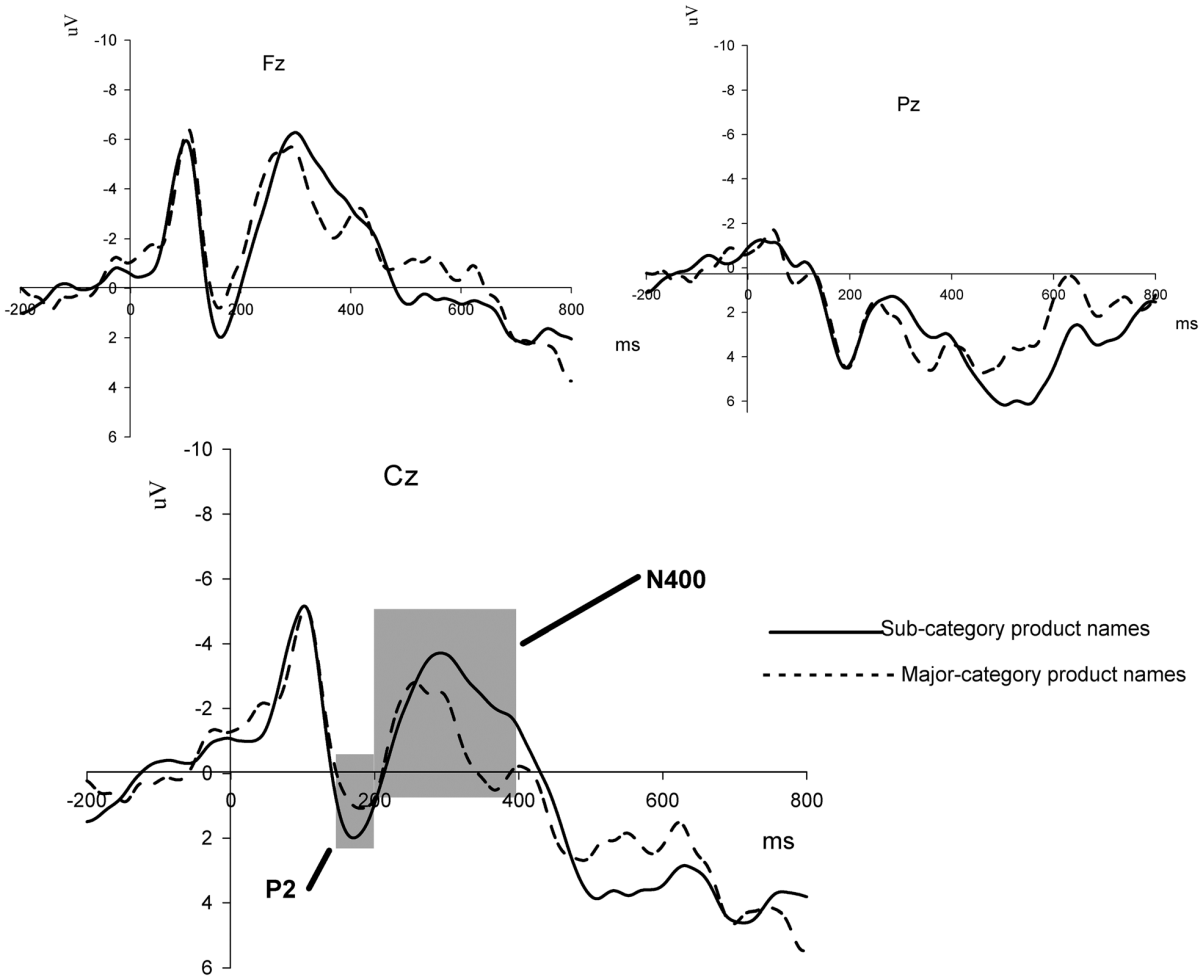
Grand-average ERPs elicited by Subcategory versus Major-category product names with prime effect of brand name at Fz, Cz, and Pz electrodes. Time window of 140 ms to200 ms for P2 quantification and time window of 200 ms to400 ms for N400 quantification marked in light gray, respectively.

The grand-average ERPs for the subcategory and major-category product names are shown in Fig. 5.

The ANOVA of the mean amplitude of P2 in the 140 ms to 200 ms time window (the raw data can be gotten from Table S3) had no significant main effect on the Subcategory and Major-category product names [*F* (1, 13)  = 3.01, *p* = 0.106]. For the mean amplitude of the N400 component in the time window of 200 ms to 400 ms (the raw data can be gotten from Table S4), the statistical result revealed a major effect for Subcategory product names (M = −1.25 µV) and Major-category product names (M = −0.16 µV) [*F* (1, 13)  = 7.03, *p* = 0.020]. Subcategory products elicited a more significantly negative N400 than Major-category products.

## Discussion

In experiment 1, we used ERP recording and neuromarketing methods to examine the unconscious processing of similarity-based categorization when consumers encounter out-of-category extensions, similar-category extensions, and in-category products with unrelated tasks. The ERP components observed in the current study, P2 and N400, should be related to two-stage categorization processes. In experiment 2, the brand extension of subcategory products elicited a more significantly negative N400 than that of major-category products, but with no significant difference in the P2 component. This result suggested that P2 reflected early low-level and similarity evaluation processing and that N400 significantly affected high-level and integrality category judgment processing in brand extension evaluation.

Most previous studies on categorization theory in consumer psychology used explicit tasks that required participants to make a classification judgment or decision based on the tasks [1],[3],[4],[41]. Experiment 1 used an implicit experimental paradigm in which participants had a no-response task but had to pay attention to stimuli and remember them for excellent performance in the test. P2 and N400 reflected brand-product categorization processing in the unrelated task paradigm.

The word pair B–H (beverage brand and household appliance) elicited significantly larger P2 (positive polarity) than B–B (beverage brand and beverage product) and B–S (beverage brand and snack product) and thus explained the low-level classification processes in the early stage by an automatic comparison of the attributes of the three categories. Many studies on mental construal have posited that the same object can be processed at different levels, ranging from low-level, concrete representations to high-level, abstract representations [42],[43]. The recorded P2 reflected the early low-level processing of stimulus classification and the engagement of attention resources in the early automatic processing of the word pair. Polezzi et al. (2008) reported that P2 distinguishes between predictable and unpredictable outcomes in economic decision making [44]. In the processing of warning signal words, there were two stages involved and P2 reflected the first stage of the early automatic cognitive process of perception and detection of hazard for the words [20]. In the experiment 1, participants processed the word pars with silent remembering as covert task, and the P2 findings suggested the early automatic detection in the semantic processes. We speculated that the P2 component might be related to the process of the participants' early detection of the product's category membership. For the soft drink brand, household appliances had made use of more cognitive resources because they were new and unpredictable and no branded products of this category were available in the market. However, beverage products as controls and snack products as similar-category products in the soft drink brand can easily be classified into one similarity-based class because of the common features of the memories of the participants, which consisted of the priming of parent brands. P2 as a perceptive ERP component reflected only the rapid and automatic assessment of the valence of the probe stimuli, and household appliances, which exceeded the expectations of the participants, attracted considerable attention. This result was supported by the suggestion that P2 reflects category identification processes [18]. However, P2 could not distinguish between beverage and snack products. The categorization process required the progressive recruitment of slow, elaborative, and semantic processing following the P2 component.

Following P2, negative component N400 recorded in experiment 1 was considered as an electrophysiological index of the elaborative classification process according to the integral category concept between S1 and S2. The soft drink brand name stimulated the memories of the participants regarding a prototype in that category, and the product of S2 was compared with the memory. Word pair B–B elicited a significantly smaller (negative-polarity) conflict than word pairs B–S and B–H. Thus, the participants unconsciously deemed that the “beverage product in S2 and beverage brand in S1 belonged to one typical product category, whereas both the snack and household appliances in S2 had some differences in attributes with the prototype of the beverage brand and cannot be classified into that category” when the attributes of the products in S2 and the brand in S1 were compared. Compared with the P2 component, N400 reflected an analytic category-based process. According to the products that emerged or did not emerge in this brand, out-of-category products as new stimuli, which were easily separated from the other two categories, elicited large P2. However, P2 could not further distinguish in-category and similar-category products by comparing the attributes. In the late stage, participants had enough time to compare the attributes of the three categories, and the conflict of B–S and B–H produced larger N400 than B–B.

In experiment 2, we further examined the relationship between ERPs and categorization processing in brand extension with subcategory and major-category product names as probe stimuli. The results provided further evidence for the two-stage categorization process in brand extension. P2 had no salient effect when subcategory products were compared with major-category products. However, the subcategory extensions elicited more significantly negative N400 than the major-category ones. In the early perceptual processing stage, the subcategory product name contained the same similarity information as the major-category product name, such as several similar attributes (e.g., sweet and small) for cookies and dessert. Therefore, P2 could not make a similarity-based distinction between major-category and subcategory products. In the late perceptual processing stage, participants gave more attention to the category information of products and then compared the attributes of the branded products and the typicality of the beverage brand. Hong and Lee (2010) suggested that people who process information at a superordinate level (general level) process conflicting ideas more inclusively and thus experience less discomfort and develop more significantly positive responses than those who process at a concrete level [45]. More conflicts were observed when the participants classified the subcategory products into the beverage brand than when they classified the major-category products. This conclusion is supported by the behavioral result that the sub-category products had a lower acceptance rate than the major-category products in experiment 2. The intense conflict in classifying the subcategory products into the beverage brand reduced the rate of acceptance of the products.

In experiment 1, no salient difference in the N400 component between the snack and household appliance categories was observed. By contrast, Ma et al. (2007) found that the component evoked by the household appliance category is more significantly negative than that evoked by the snack category [46]. The main reason for these conflicting results may be the differences in the task-oriented manipulation in the two studies. Experiment 1 used an implicit task in which participants were required to remember S1 and S2, whereas Ma et al. used an explicit task in which participants were asked to judge whether brand extension (S1–S2) was suitable. The unrelated task occupied some attention resource so that inadequate attention resource may be available to distinguish the household appliance category from the snack category. In Ma et al., however, the task was related to brand extension, and the participants could exert all effort to perceive and make judgments regarding the product. Different intense conflicts may occur when household appliance or snack categories are classified into a beverage brand. The decision-making-related task also evoked large P300, which might overlap with N400.

## Conclusions

In this study, we used a prime–probe paradigm in two experiments and investigated the two-stage categorization process with stimuli related to brand extension. The P2 of ERP can reflect the early low-level and similarity-based processing of the first stage, and N400 can reflect the later analytic and category-based processing of the second stage. In the first stage, beverage brands as prime stimuli demonstrated the association of the participants between brand-related typical products and their attributes, and household appliances as out-of-category products (new stimuli) were easily classified from the other two categories. The P2 component reflected a low-level categorization process. In the second stage, participants had time to execute an elaborative and complicated process and distinguished similar-category products (snack category) from typical products (beverage category). This distinction was reflected by the N400 component. From another perspective of categorization theory, we further validated that the holistic category concept formed in the second stage.

## Supporting Information

Table S1 **The data of P2 in the experiment 1.** It includes the mean amplitude in the time window of 150 ms to 250 ms for three categories (Beverage, Snack and Household appliance) at F3, Fz, F4, FC3, FCz, FC4, C3, Cz and C4 electrodes.
(XLSX)

Table S2 **The data of N400 in the experiment 1.** It includes the mean amplitude in the time window of 300 ms to 450 ms for three categories (Beverage, Snack and Household appliance) at F3, Fz, F4, FC3, FCz, FC4, C3, Cz, C4, CP3, CPz, CP4, P3, Pz and P4 electrodes.
(XLSX)

Table S3 **The data of P2 in the experiment 2.** It includes the mean amplitude in the time window of 140 ms to 200 ms for subcategory and major-category product names at F3, Fz, F4, FC3, FCz, FC4, C3, Cz and C4 electrodes.
(XLSX)

Table S4 **The data of N400 in the experiment 2.** It includes the mean amplitude in the time window of 200 ms to 400 ms for subcategory and major-category product names at F3, Fz, F4, FC3, FCz, FC4, C3, Cz, C4, CP3, CPz, CP4, P3, Pz and P4 electrodes.
(XLSX)
